# S35. NEURORADIOLOGICAL FINDINGS IN CHILD AND ADOLESCENT PATIENTS WITH PSYCHOTIC DISORDERS

**DOI:** 10.1093/schbul/sby018.822

**Published:** 2018-04-01

**Authors:** Justo Pinzón Espinosa, Adriana Fortea, Laura Espinosa, Anna Giménez, Gerard Anmella, Immaculada Baeza, Gisela Sugranyes

**Affiliations:** 1 Hospital Clinic of Barcelona; 2 Hospital Clínic of Barcelona, IDIBAPS

## Abstract

**Background:**

A 22 - 31% prevalence of abnormal radiological findings (RF) has been reported among patients with first episode of psychosis (FEP), ranging from clinically non-significant findings to overt neurological pathology. While one study (Borgwardt et al., 2006) found a higher proportion of RF in adult subjects at high risk for psychosis (35%) and FEP patients (40%) than in patients with depression (18%) or healthy controls (12%), another found a similar increase in RF in patients with affective and psychotic disorders (Landin et al, 2016). This suggests that macroscopic brain anomalies may be characteristic of at least a subset of patients in the early stages of psychosis, and these RF may not be specific to schizophrenia, but also to psychosis with affective symptoms.

To this day, all published research studies have been done in primarily adult samples. Psychosis with onset before age 18 may be associated with more salient biological features linked with greater genetic load and neurodevelopmental antecedents (Arango, 2014).

**Aims:**

To assess the prevalence of neuroradiological abnormalities in a population with early-onset psychosis (EOP) in comparison to a sample of community controls, and to evaluate the association of these findings with the type of psychotic disorder of the patients.

**Methods:**

Design: Naturalistic, observational, retrospective, single-center controlled study.

A chart review of individuals admitted to the Inpatient unit of the Dept. of Child and Adolescent Psychiatry and Psychology from January 2013 to December 2016 was done. Patients were 6 to 17 years old, fulfilled DSM-IV-TR criteria for a psychotic disorder (PD), and had a radiology report of a brain MRI. The community control (CC) group had a similar age and gender distribution and no current diagnosis of any psychotic disorder. Any neurological or severe medical condition or head trauma with loss of consciousness were exclusion criteria for both groups.

Sociodemographic, clinical, and radiological variables were recorded for both groups. Given the association of abnormal RF with prematurity, perinatal complications and neurodevelopmental disorders, these data were collected and sorted dichotomously.

Descriptive statistical analysis consisted of a means and standard deviation for quantitative variables and percentages for qualitative variables. Between-group differences were calculated with chi-square test or Fisher’s test using IBM SPSS v23.

**Results:**

A total of 191 individuals were included (127 PD vs 64 CC, mean ages: 14.7 ± 1.8 vs 13.8 ± 2.3, t=3.0, p=.01; %females: 55.9.0% vs 60.9%, χ2=.44, p=.50). Main diagnoses in PD were psychosis not otherwise specified (PNOS) (59.1%), schizoaffective disorder (SAD) (12.6%), schizophrenia (SCZ) (11.0%), bipolar disorder (BD) (8.7%) and major depression with psychotic features (MDD) (8.7%).

The PD group presented with a significantly higher prevalence of qualitative neuroimaging abnormalities in comparison to CC (21.3% (n=27) vs 6.2% (n=4), χ2=7.1, p=.008). These included arachnoid cysts, dilated perivascular space or white matter intensity anomalies. The prevalence of abnormal RF was 25.3% in PNOS, 21.4% in SCZ, 18.2% in MDD 12.5% in SAD and 9.1% in BD.

**Discussion:**

A significantly higher prevalence of RF was found in youth with both affective and non-affective psychosis compared to similar-aged controls, concurring with some (Borgwardt et al., 2006; Landin et al., 2016), yet not with other (Sommer et al., 2013) observations in adult samples. These findings may reflect an impact of subtle biological alterations associated with psychosis on brain development, which may be more salient in early-onset cases.

Our data highlight the need to continue assessing the significance of abnormal RF in patients with EOP

